# Exosome CTLA-4 Regulates PTEN/CD44 Signal Pathway in Spleen Deficiency Internal Environment to Promote Invasion and Metastasis of Hepatocellular Carcinoma

**DOI:** 10.3389/fphar.2021.757194

**Published:** 2021-10-20

**Authors:** Yongdan Wang, Pan Li, Shuai Mao, Zhuomao Mo, Zhirui Cao, Jin Luo, Meiling Zhou, Xifeng Liu, Shijun Zhang, Ling Yu

**Affiliations:** ^1^ Department of Traditional Chinese Medicine, The First Affiliated Hospital, Sun Yat-sen University, Guangzhou, China; ^2^ Second Clinical College, Guangzhou University of Chinese Medicine, Guangzhou, China; ^3^ AMI Key Laboratory of Chinese Medicine in Guangzhou, Guangdong Provincial Hospital of Chinese Medicine, Guangzhou, China; ^4^ School of Life Sciences, Xiangya Medical College, Central South University, Changsha, China

**Keywords:** hepatocellular carcinoma, spleen deficiency, exosome, CTLA-4, PTEN

## Abstract

Hepatocellular carcinoma (HCC) is one of the most common primary cancers, and its pathogenesis is complicated and difficult to screen. Currently, there is no effective treatment. In traditional Chinese medicine, a large proportion of patients with HCC have been diagnosed with spleen deficiency (SD) syndrome and treated with tonifying traditional Chinese medicine, which has significant clinical efficacy. However, the role and molecular mechanism of SD in HCC remain unclear. In this study, 40 mice were randomly divided into four groups: control, SD, HCC, and SD-HCC groups. The liver cancer model of SD was established by reserpine induction and orthotopic transplantation. The effects of SD on the proliferation, apoptosis, invasion, and metastasis of HCC cells were studied by cell proliferation, cell apoptosis, cell scratch, and transwell assay. We found that compared with the HCC group, the protein expressions of cytotoxic T lymphocyte antigen 4 (CTLA-4), programmed cell death protein 1 (PD-1), phosphatase and tensin homolog (PTEN), and AKT (also known as protein kinase B or PKB) in the exosomes of the SD-HCC group were upregulated. In addition, the metastases and self-renewal of exosomes in the SD-HCC group were more aggressive than those in the HCC group, which could be partially reversed with the addition of CTLA-4 inhibitors. Further studies showed that in the internal environment of SD, CTLA-4 promoted tumor invasion and metastasis by regulating the PTEN/CD44 pathway. In conclusion, our findings suggest that during SD in the internal environment, exosome CTLA-4 regulates the PTEN/CD44 signal pathway to promote the proliferation, self-renewal, and metastasis of liver cancer.

## Introduction

Hepatocellular carcinoma (HCC) is a highly prevalent and lethal cancer, ranking the fifth most commonly diagnosed cancer and the third leading cause of cancer-associated deaths worldwide (Chen et al., 2016). The incidence of HCC has increased significantly recently, with about 840,000 new cases each year (Bray et al., 2018). It has been reported that the incidence and mortality of HCC in China account for 50% of HCC in the world (Feng et al., 2019). Hepatitis B virus (HBV) infection is the main cause of HCC in East Asia, especially in China (Bray et al., 2018). The rapid development and high recurrence are major challenges in the treatment of HCC due to its highly metastatic nature (Gish et al., 2007; Hu et al., 2020). Therefore, it is urgent to seek valuable biomarkers and potential therapeutic targets to improve the clinical curative effect on HCC patients.

The imbalance of the internal environment is closely related to the occurrence and development of tumors (Craig et al., 2020; Llovet et al., 2021). Generally, only about 5% of tumors could be explained by genetic factors alone, whereas most tumors are the result of a combination of environmental and genetic factors (Bhattacharjee et al., 2013). In the TCM (traditional Chinese medicine) therapy systems, “Bian Zheng Lun Zhi” is the core, that is, a treatment based on syndrome differentiation, which prioritizes to adjust the internal environment (Zhang et al., 2020). TCM syndromes are the characteristics of all syndromes in a patient's clinical manifestations essentially and help guide the design of individualized treatments (Ji et al., 2016). In TCM theory, symptoms such as loose stools, abdominal distension after meals, loss of appetite, sallow complexion, weight loss, general weakness, and/or low disease resistance could be summarized as spleen deficiency (SD) (Luo et al., 2017). Interestingly, the majority of patients with cancer cachexia have been diagnosed with SD in China (Sun et al., 2016a; Zhang et al., 2020). According to the literature, SD may lead to dysfunction in T cell recognition by disordering the expression of TCRVβ in rats (Chen et al., 2014). In addition, another study reported that immunoregulatory traditional Chinese medicine is beneficial to liver cancer and induces cell apoptosis through the Caspase-3/PARP signaling pathway (Yu et al., 2021). The above studies suggest the importance of the internal environment of SD in the occurrence and development of immune recognition disorders of HCC.

The barrier to successful cancer immunotherapy is the capability of tumors to escape from the host’s immune system (Whiteside et al., 2011). Exosomes, small membranous sacs of endocytic origin (30–150 nm), are considered as intercellular messengers that can carry a large number of macromolecular cargos, including proteins, mRNA, lipids, and miRNA (Wen et al., 2016; Bach et al., 2017; Ruivo et al., 2017; Su et al., 2021). Studies have found that exosomes derived from tumors carry immunosuppressive proteins, including PD-1, CTLA-4, FasL, TRAIL, CD39, and CD73, which induce apoptosis and depletion of T lymphocytes to achieve tumor immune escape (Whiteside, 2013; Ukrainskaya et al., 2019; Benecke et al., 2021). Cytotoxic T lymphocyte antigen 4 (CTLA-4), a member of the immune protein superfamily, competes with the T cell co-stimulator CD28 for binding to CD80 and CD86 with a higher affinity to antigen-presenting cells during the priming phase in the lymph nodes and conveys inhibitory signals within the T cells (Van Coillie et al., 2020; Goenka et al., 2021). Clinical evidence has shown that anti–CTLA-4 therapy can enhance the activation of effector T cells (Maker et al., 2005), increase the ratio of effector T cells to Treg (Quezada et al., 2006; Curran et al., 2010; Ou et al., 2018), and promote the transport of activated T cells to tumor tissues (Quezada et al., 2006). Our previous study found that traditional Chinese medicine could improve the tumor microenvironment of patients to treat liver cancer and speculated that the immune checkpoints CTLA-4, LAG-3, and BIRC5 are the key targets for activating immune cells to treat liver cancer (Mo et al., 2020). However, only a minority of people can respond to immunotherapy (Mahoney et al., 2015). Consequently, it is still a critical challenge to explore the molecular mechanisms of immunotherapy for HCC.

PTEN is known as a tumor suppressor protein in most tumors, which is located in Chromosome 10 (Su et al., 2016; Luongo et al., 2019). The loss of PTEN function due to epigenetic silencing or genetic aberration has been associated with malignant transformation, progression, chemotherapy response, and survival of a variety of cancers (Wang et al., 2018a; Raffone et al., 2019). PTEN regulates PI3K/Akt/mTOR by its phosphatase activity, which is one of the most important signaling pathways for cell growth and survival in cancer (Su et al., 2016; Lee et al., 2018; Luongo et al., 2019). It has been reported that CD44 is one of the biomarkers and a key regulator of cancer stem cells (CSCs), including self-renewal, tumor initiation, and metastasis (Wang et al., 2018b). PTEN plays a critical role in the immobility and regulation of the cell cycle entry of transforming stem cells (SCs), and the loss of PTEN tends to promote cancerous phenotypes (Luongo et al., 2019). It has been found that miR-21, a gene known to target PTEN, significantly regulates the growth and/or differentiation of CSCs in colon cancer (Yu et al., 2015). The imbalance of the PTEN/CD44 signaling pathway plays a vital role in maintaining the characteristics of CSC (Luongo et al., 2019; Cetintas and Batada, 2020). Nevertheless, the link between CTLA-4 and PTEN/CD44 signaling pathway has not been reported.

**SCHEME 1:**
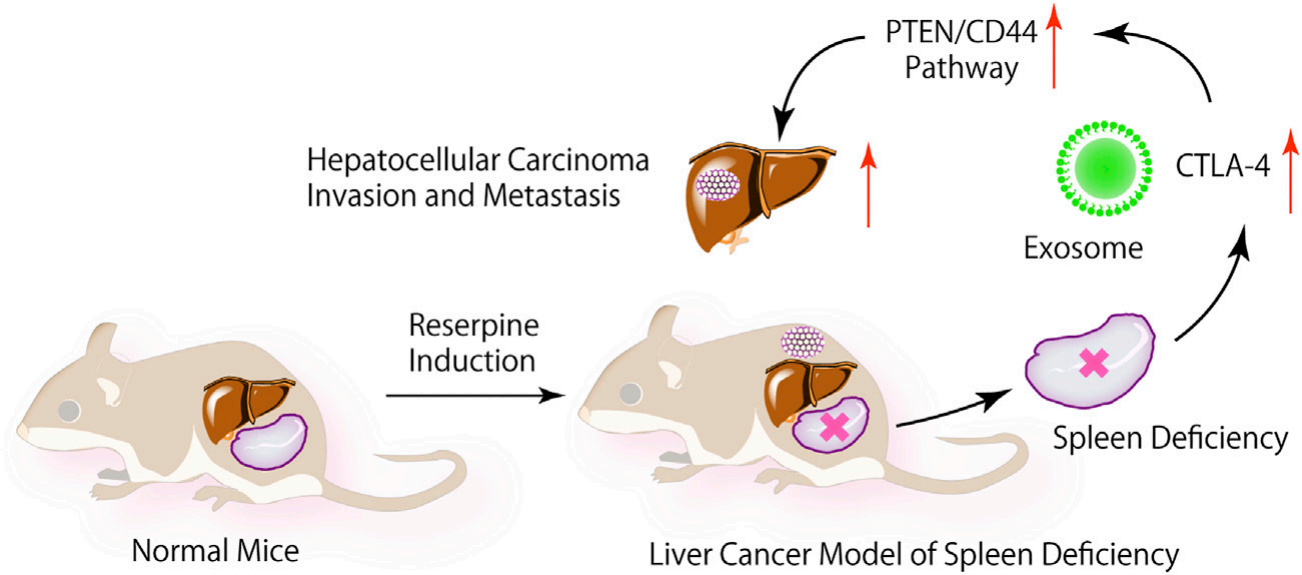
In the internal environment of SD, exosome CTLA-4 regulate the PTEN/CD44 signal pathway to promote the proliferation, self-renewal, and metastasis of liver cancer.

Based on the above studies, we proposed the hypothesis that the change of CTLA-4 expression in exosomes of SD internal environment is related to the PTEN/CD44 signaling pathway, as demonstrated in Scheme 1. Exosome CTLA-4 is an important factor in HCC. Therefore, we aimed to explore the underlying mechanism of exosome CTLA-4 in HCC progression. We have tried our best to seek a new therapeutic target for patients with liver cancer.

## Materials and Methods

### Chemicals and Materials

Reserpine powder (R817202), purity greater than 98.0%, was purchased from Jiusuo Technology Co. Ltd. (Guangzhou, China). Glacial acetic acid (B018) was purchased from Feng Wei Technology Co. Ltd. (Guangzhou, China). The preparation process of the reserpine solution is as follows: 10 mg of reserpine powder was added with 10 ml acetic acid to obtain 1 mg/ml reserpine solution A and stored at 4°C. Before each experiment, 0.1 ml reserpine solution A and 9.9 ml sodium chloride as injection were added to obtain 0.01 mg/ml reserpine solution B. In addition to special labeling, other chemicals were purchased from UZhiYi Biotechnology Co. Ltd. (Guangzhou, China).

### Cell Line and Cell Culture

Mouse HCC cell line hep1-6 was obtained from the Institute of Biochemistry and Cell Biology (Shanghai, China). The cell line was cultured in RPMI-1640 medium (Hy Clone, Los Angeles, CA, United States), which contained 10% fetal bovine serum (FBS) and 5% carbon dioxide. The ambient temperature was controlled at about 37°C. HCC cell lines were identified by microsatellite analysis, in which the cells were cultured for no more than 2 months.

### Animals and Groups

Forty male SPF C57BL/6 mice (4 weeks old, 18–20 g) and three male SPF BALB/C-Nu (4 weeks old, 16–18 g) were purchased from GemPharmatech Co. Ltd. (Jiangsu, China). Laboratory animal quality certificate no: SCXK(SU)2018-0008. The animals were kept in a specific pathogen-free environment with a temperature of 25°C, relative humidity of 60–80%, free eating, and drinking water. All experiments were conducted in the SPF laboratory of the animal center of the first affiliated hospital, Sun Yat-Sen University (Guangzhou, China). The license number was SYXK (Guangdong) 2020-0108. Experimental animals were used in accordance with the 3R principle for humane care. The animal experiments were approved by the clinical research and animals ethics committee of the first affiliated hospital, Sun Yat-Sen University. In the SPF barrier environment, 40 C57BL/6 male mice were randomly divided into the normal group, SD group, liver cancer group, and SD liver cancer group. There were 10 mice in each group and 40 mice were labeled.

### The Establishment of Spleen Deficiency Model

A mouse model of SD was established by reserpine. Mice in the SD group and SD liver cancer group were subcutaneously injected with reserpine solution B, one time per day at a dose of 0.1 ml/10 g. At the same time, the normal and liver cancer groups were subcutaneously injected with sodium chloride once per day at the same dose of 0.1 ml/10 g for 14 consecutive days. The weight of all the mice was measured daily, and the amount of food they ate in each cage was recorded. At the same time, the odor, mental state, body cold and heat, respiratory status, hair color, food intake, and stool of all mice in the four groups were observed. At the end of the 14th day, the severity of SD in mice was evaluated by using the SD rating scale (Table 1).

**TABLE 1:** Spleen deficiency rating scale.

Item	One point	Two-point	Three-point	Four-point
Odor	No unusual	Faint odor	Serious odor	Extremely odor
Mental state	Normal	Tiredness	Drowsy	Irritability
Body cold and heat	Normal	Crouch	Serious cold	Extremely cold
Respiratory status	Normal	Pant	Shortness of breath	Faint breath
Hair color	Normal	No bright	Shaggy hair	Rarer and yellow
Stool	Normal	Slight wettability	Serious wettability	Extreme wettability
Food intake	Normal	Reduce food intake by 50%	Reduce food intake by 75%	Barely food intake

The calculated sum of the scores of each item (total score ≤ 6, no SD; 7 ≤ total scores ≤ 12, mild SD; 13 ≤ total scores ≤ 18, typical SD; 19 ≤ total scores ≤ 24, severe SD).

The mice were forced to swim, and the time from the beginning of swimming to the first stop swimming was recorded, which is helpful to judge the effect of the SD model in mice. In this case, a water tank with a smooth sidewall and a suitable size was prepared with water temperature kept at 25°C. First, the mice were put into a water tank for 30 s, and then the mice were acclimated to the water tank environment. The mice that could not swim or could not swim very well were excluded, and the order of the mice that were put into the water tank was recorded. After the mice returned to their normal state, they were placed in the tank again in the same order as before, and the total duration of swimming–struggling–floating immobility was recorded and a statistical analysis of the results performed.

### BALB/C-Nu Mice Tumorigenesis Experiment

When C57BL/6 mice were induced with SD for 1 week, precultured hepa1-6 cell suspension (1 × 10^7^/ml) was subcutaneously injected into the armpit of BALB/C nude mice at 0.1 ml. Each mouse was inoculated with 1 × 10^6^ cells. The mental status and tumorigenesis of the nude mice were observed every day. When the diameter of the subcutaneous tumor was about 1 cm at 2 weeks, the tumor could be used to establish the liver cancer model.

### Establishment of Liver Cancer Model

One week after the SD model was established, the orthotopic liver cancer models were established in the liver cancer group and the spleen liver cancer group. Mice were anesthetized with 1% pentobarbital sodium solution (50 mg/kg). At the same time, the BALB/C nude mice were killed by cervical dislocation, the tumor was quickly removed under aseptic conditions, the necrotic tissue was removed, and the flesh-like tissue was cut into 1-mm^3^-sized pieces in PBS solution. The skin was incised at the xiphoid process and the left lobe of the liver pulled out of the abdominal cavity. The 1-mm^3^-sized tumor tissue was placed in the cannula (5 mm from the tip of the cannula), and the cannula was inserted into the liver surface at an angle of 10 degrees, then the tumor tissue was implanted under the liver capsule. Absorbable gelatin sponge was applied to the bleeding area, and the liver lobe was sent back to the abdominal cavity. Penicillin solution was used to flush the abdominal cavity, and absorbable suture was used to close the abdominal cavity. The mice were closely monitored for survival and weighed daily. After the liver cancer model was established for 28 days, blood and tissue samples were collected.

### Extraction of Exosomes

The serum exosomes of mice were extracted by ultracentrifugation. The blood (500 µL) was collected in a centrifuge tube, then centrifuged at 4°C for 5 min at 3,000 r/min after the blood had coagulated. The supernatant serum was transferred to a new centrifuge tube and centrifuged at 4°C for 10 min at 300 × *g*. The supernatant was centrifuged at 4°C for 10 min at 2000 × *g*, then the precipitates were discarded, and the supernatant was further centrifuged for 70 min at 10000 × *g*. After this step, the supernatant was transferred to a new high speed centrifuge tube, centrifuged at 4°C for 70 min at 100,000 × *g*. The supernatant was discarded and added with the appropriate amount of PBS buffer to make the exosomes suspended in the PBS buffer. The suspension was centrifuged at 4°C for 70 min at 100,000 × *g*, and the supernatant was discarded. The bottom precipitated exosomes were suspended in 200 μL of 4°C precooled PBS buffer and kept at −80°C for future usage.

### MTT Assay

HepG2 cells were digested with 0.25% trypsin at the logarithmic growth stage, digestion was stopped with serum-containing medium, and these were then beaten into a single-cell suspension, stained using trypan blue stain, and counted. The cells were inoculated into a 96-well plate, and another blank control well was set up, which was only adding a complete culture medium, and cultured in the incubator (37°C, 5% CO_2_, saturation humidity) for 24 h. The cells were cultured to form a monolayer covering the bottom of the well. The culture solution was then removed and added with 10 μL PBS (control group), serum exosomes of liver cancer, serum exosome of liver cancer with SD, and serum exosome of liver cancer with SD that combined with 50 ng/ml CTLA-4, 100 ng/ml CTLA-4, or 200 ng/ml CTLA-4 inhibitor. After culturing at 37°C for 72 h, 50 μL (1 mg/ml) of MTT was added and cocultured for 4 h, then the supernatant was discarded and replaced with 150 μL DMSO solution. The plate was placed in the microwell plate oscillator to oscillate for 15 min, and the blue-purple crystal dissolved completely. The OD values of each well were measured at 570 nm wavelength by enzyme-linked immunosorbent assay. Each group was tested with 12 repeating wells.

### Apoptosis Assay

Apoptosis of HepG2 cells was detected by Annexin V–FITC/PI double-labeled flow detector. The cells were cultured in a DMEM medium containing 10% fetal bovine serum (FBS) at 37°C, 5% CO_2_, and saturated humidity for the logarithmic phase. The cell concentration was adjusted to 1 × 10^4^/ml. The cells were inoculated into a six-well cell plate with each well about 2 ml. Then, added with 10 µL PBS (control group), serum exosomes of liver cancer, serum exosome of liver cancer with SD, and serum exosome of liver cancer with SD that combined with 200 ng/ml CTLA-4 inhibitor. According to the instructions of the Annexin V–FITC/PI double staining kit, the cells were collected 24 h later, digested by trypsin, then centrifuged to collect the cells. After resuspending the cells with 1 ml precooled PBS, 100 μL of cell suspension solution was transferred to a 5-ml culture tube and added with 5 μL Annexin V–FITC and 5 μL PI. After incubation at room temperature for 15 min in the dark, the cells were added with 400 μL of combined buffer and analyzed by flow cytometry.

### Cell Migration Assay

HepG2 cells (HepG2, HepG2 + HCC exosome, HepG2 + SD-HCC exosome, HepG2 + SD-HCC exosome + CTLA-4 inhibitor) were selected in the logarithmic phase. The cells were digested with trypsin, centrifuged, and washed twice with PBS. The concentration of cells was adjusted to 2 × 10^5^/ml in a serum-free medium. The transwell chamber was placed in a 24-well plate, 100 μL cell suspension was added in the transwell chamber, then along with 5 µL PBS (control group), serum exosomes of liver cancer, serum exosome of liver cancer with SD, and serum exosome of liver cancer with SD that combined with 200 ng/ml CTLA-4 inhibitor were added. 600 μL medium containing 10% FBS was added in the lower chamber and incubated for 24 h at 37°C in 5% CO_2_. After removing the transwell chamber and discarding the culture fluid from the well, it was rinsed with PBS and 4% paraformaldehyde for 30 min. The chamber was dried properly and dyed with crystal violet for 30 min, using a cotton swab to gently remove the non-penetrating cells from the surface of the membrane, and washed twice with PBS. Under the microscope, five high-power (×400) views were randomly selected to observe the cells in each group. The number of transmembrane cells was used to express the ability of locomotion. Cells of each group were set into three double wells.

### Cell Invasion Assay

A serum-free medium containing fibronectin was added to the lower chamber of the transwell cell membrane. At 4°C, Matrigel was melted overnight, diluted in the serum-free medium, and applied to the upper surface of the transwell chamber at an amount of 100 μL Matrigel (1 mg/ml) per well. This was set in a 37°C incubator for 30 min. The other experimental steps and cell processing methods were the same as the Cell Migration Assay. The invasive ability was expressed by the number of transmembrane cells. The experiment was repeated three times.

### Wound-Healing Assays

Lines were drawn across the back of the six-well plate, and 5 × 10^5^ cells were inoculated into each well. The cell processing methods are the same as the Apoptosis Assay. On the next day, the back of the six-well plate was scratched using the tip of a pipette. The wells were then washed three times with sterile PBS to remove the cells below the underline to ensure that the remaining space was clearly visible. Then, the serum-free medium was replaced, and the cells were cultured in a 5% CO_2_ incubator at 37°C. At 0, 24 h, and 48 h, the wounds were observed and photographed under a microscope. The width of the wounds at each time was recorded.

### H&E Staining of Tissue Sections

The liver tissues of the normal group, SD group, liver cancer group, and SD liver cancer group were selected. After the tissue was fixed with 10% formaldehyde, it was put into alcohol ranging from low-concentration alcohol to high-concentration alcohol for dehydration treatment. The tissue block was immersed in xylene for transparency and then embedded in paraffin. The paraffin wax was removed with xylene before staining. Then the slides were washed through the high concentration to low concentration of alcohol, and finally immersed into distilled water. The slices were dyed in hematoxylin solution for 5 min, flushed with water for 1 hour, then dehydrated with 70 and 90% alcohol for 10 min and stained by eosin for 2 minutes. After staining, the slides were dehydrated with alcohol from low concentration to high concentration and then made transparent by using xylene. Finally, the slices are sealed, labeled, and set aside. The histomorphological structure was observed under the microscope.

### Immunohistochemical Assay

The tissue was fixed using 10% formaldehyde, dehydrated from low concentration alcohol to high concentration alcohol, made transparent using xylene, embedded in paraffin, and sectioned. The tissue slices were placed in the oven at 60°C for 30 min, and then were taken out, cooled, dewaxed, and hydrated with high concentration alcohol to low concentration alcohol, which were then was incubated with 3% hydrogen peroxide at room temperature for 5–10 min. The slices were repaired by microwave in 0.01 mol/L citric acid buffer (pH = 6.0) for 30 min and sealed with 5% normal sheep serum for 30 min at room temperature. The sheep serum was removed from the section and added to the diluted primary antibody (1:250). Then, this was incubated overnight at 4°C. The diluted secondary antibody was added and incubated at 37°C for 30 min. This was then rinsed with PBS for 2 min (three times). A moderate amount of *Streptomyces* antibiotic protein peroxidase was added and was then incubated at 37°C for 30 min and rinsed with PBS for 3 min (two times). These were then stained with DAB color for 5 min and rinsed thoroughly with distilled water. Restaining, dehydration, transparency, and sealing were performed. Five high-power microscopic views were randomly selected from each section to observe the staining of the cells.

### Western Blotting Assay

Firstly, liver tissue of the normal group and SD group, and liver cancer tissue of the liver cancer group and SD liver cancer group were extracted. The tissues were split with RIPA for 30 min, and then the split solution was transferred to a centrifuge tube and centrifuged for 10 min at 4°C, 12,000 rpm. The supernatant (protein sample) was packed in a 0.5-ml centrifuge tube and stored at −20°C. The concentration of protein was determined by the BCA (bicinchoninic acid) method.

After the addition of 20 g of the sample, the protein was separated by 10% SDS-PAGE and transferred to the PVDF membrane by the wet transfer membrane. When the membrane transfer is completed, the membrane is cut into different bands according to the target protein molecule of the target protein. Sealing fluid containing 5% skimmed milk powder was used to seal this for 2 h at room temperature. The diluted (1:1000) primary antibody (CTLA-4, CD80, CD86, PD-1, PD-L1, PTEN, CD44, and β-Actin) was added and rested at 4°C overnight. It was then rinsed with TBST three times (10°min/time). An HRP-labeled anti-rabbit or anti-rat IgG was used as the secondary antibody (1:10000) and incubated at room temperature for 1 hour. This was rinsed again with TBST for three times (10 min/time). Finally, chemiluminescence detection (light-proof operation) was performed, and β-actin was used as the internal control. The ratio of various proteins to β-actin indicates the relative expression level of each protein.

### Real-Time Quantitative PCR Analysis

For liver tissue of the normal group and SD group, liver cancer tissue of liver cancer group and SD liver cancer group, the total RNA was extracted according to the instructions of the TRIzol RNA extraction kit. RNA concentrations were measured using an ultraviolet photometer, and mRNA was reverse-transcribed into cDNA by reverse transcription kit then stored at −20°C after the reaction. GAPDH was used as the internal control for the relative quantitative analysis. Then, PCR amplification was performed using cDNA as a template. The total reaction system of real-time PCR was 20 L. The amplification procedures were pre-denaturation in 95°C, 2 min; denaturation in 95°C, 20 s; annealing in 60°C, 20 s; and extension in 72°C, 30 s, altogether 40 cycles. At 72°C for 10 min after the last cycle, the relative expression levels of CTLA-4, PD-1, PD-L1, PTEN, and CD44 mRNA were analyzed by the 2^−△△Ct^ analytical method. The experiment was repeated three times independently. Genespecific primers are listed at supplementary material.

### Statistical Analysis

SPSS 26.0 software was used for data analysis and Prism GraphPad 5.0 software was used for mapping and measurement. The data are expressed as mean ± standard deviation. The differences between the two groups were tested by Student's t-test, and the differences among many groups were compared by variance analysis. Groups tested with *p* < 0.05 showed that the difference was statistically significant. All the tests were repeated three times independently.

## Results

### Establishment of the SD-HCC Model With Reserpine

**FIGURE 1:**
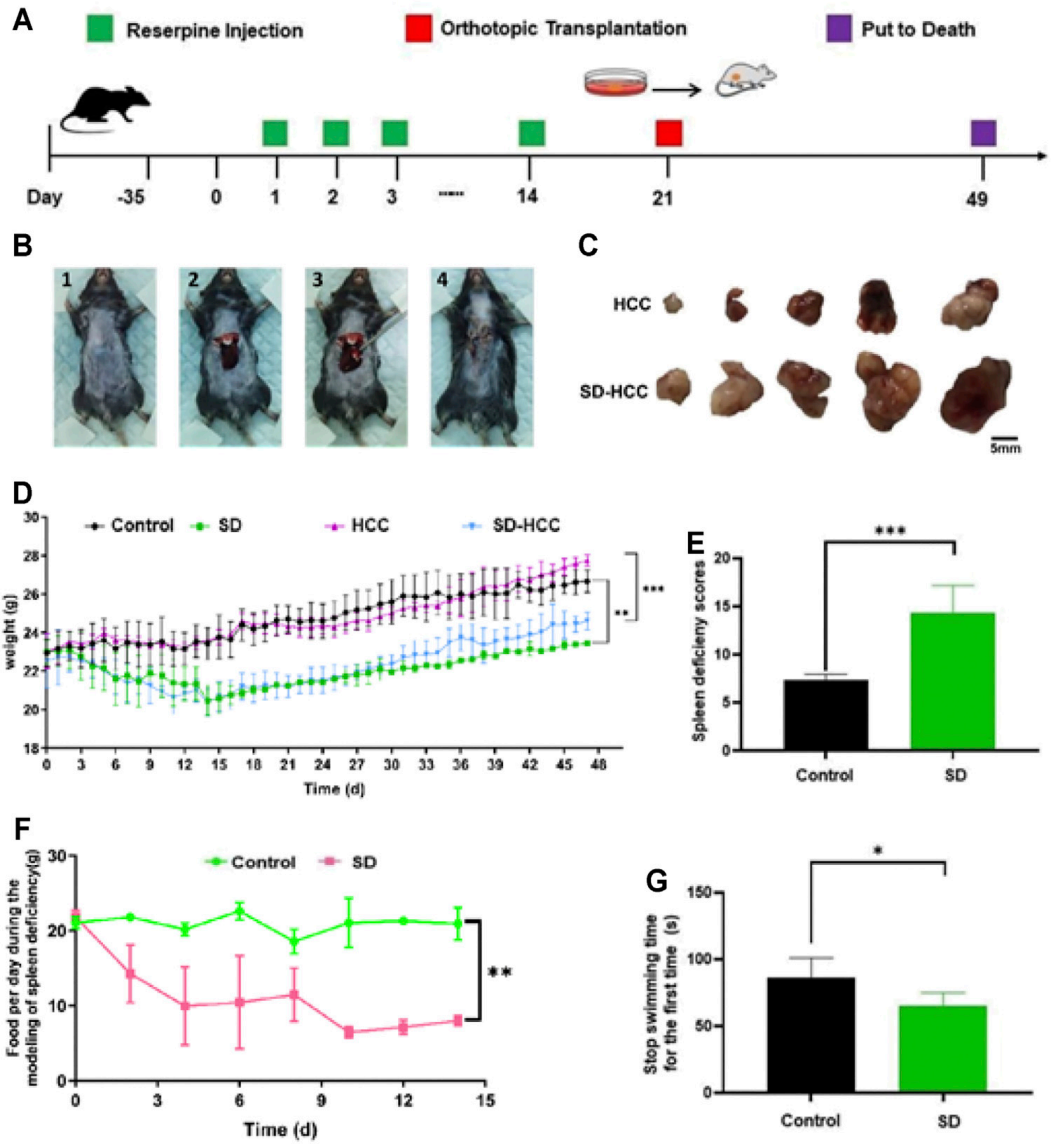
Preparation of orthotopic transplantation liver cancer model with SD. **(A)** Flow chart; **(B)** transplantation process: 1) shave and disinfect the surgical area, 2) squeeze out the liver lobe, 3) inoculate the tumor mass, 4) suture the wound; **(C)** comparison of tumor size; **(D)** bodyweight change curve; **(E)** SD score statistics; **(F)** changes in the diet of mice during the preparation of SD model; **(G)** stop swimming time statistics. *n* = 3, *p < 0.05, **p < 0.01, ***p < 0.001.

After passing the quarantine period, the mice were treated with reserpine (0.1 mg/kg) daily for 14 days. Orthotopic liver cancer transplantation was performed 14 days later, and the specimens were collected after 49 days. The flow chart is shown in Figure 1A. The SD score (Luo et al., 2017) is an important method for assessing SD by evaluating scores for factors including body odor, mental state, chill and fever, respiration, fur, feces, and appetite. The SD scores assessment showed that the SD group had lower scores than the control group (*p* < 0.001) (Figure 1E). Compared with the control group and HCC group, the weight of the mice in the SD-HCC and SD groups was lower and significantly different (*p* < 0.01) during 5–49 days (Figure 1D). The SD group consumed less food per day than the control group (Figure 1F). The forced swim test revealed that the stop swimming time of the SD group was shorter for the first time (Figure 1G). The preparation process of orthotopic transplantation liver cancer model is as follows: first, mouse HCC hepa1-6 cells were inoculated subcutaneously into nude mice. When a 1 cm^3^ tumor was formed, the tumor was taken out and cut into 1-mm^3^-sized pieces for use. The operation area in the C57BL/6 black mouse was shaved and disinfected. A 0.5–0.8 mm incision was made near the breastbone, and the liver was extruded. The spare tumor tissue was inoculated into the liver lobe with a trocar. After pressing the hemostatic, the liver lobe was returned to the body, and the incision was sutured. The animals were dissected 28 days after the operation. The liver tumor grew well, and the tumor size of the mice in the HCC group was significantly smaller than that in the SD-HCC group, which demonstrated that the orthotopic transplantation model was successfully prepared. The transplantation process and tumor size comparison pictures are shown in Figures 1B,C.

### Exosomes Extraction and Verification

**FIGURE 2:**
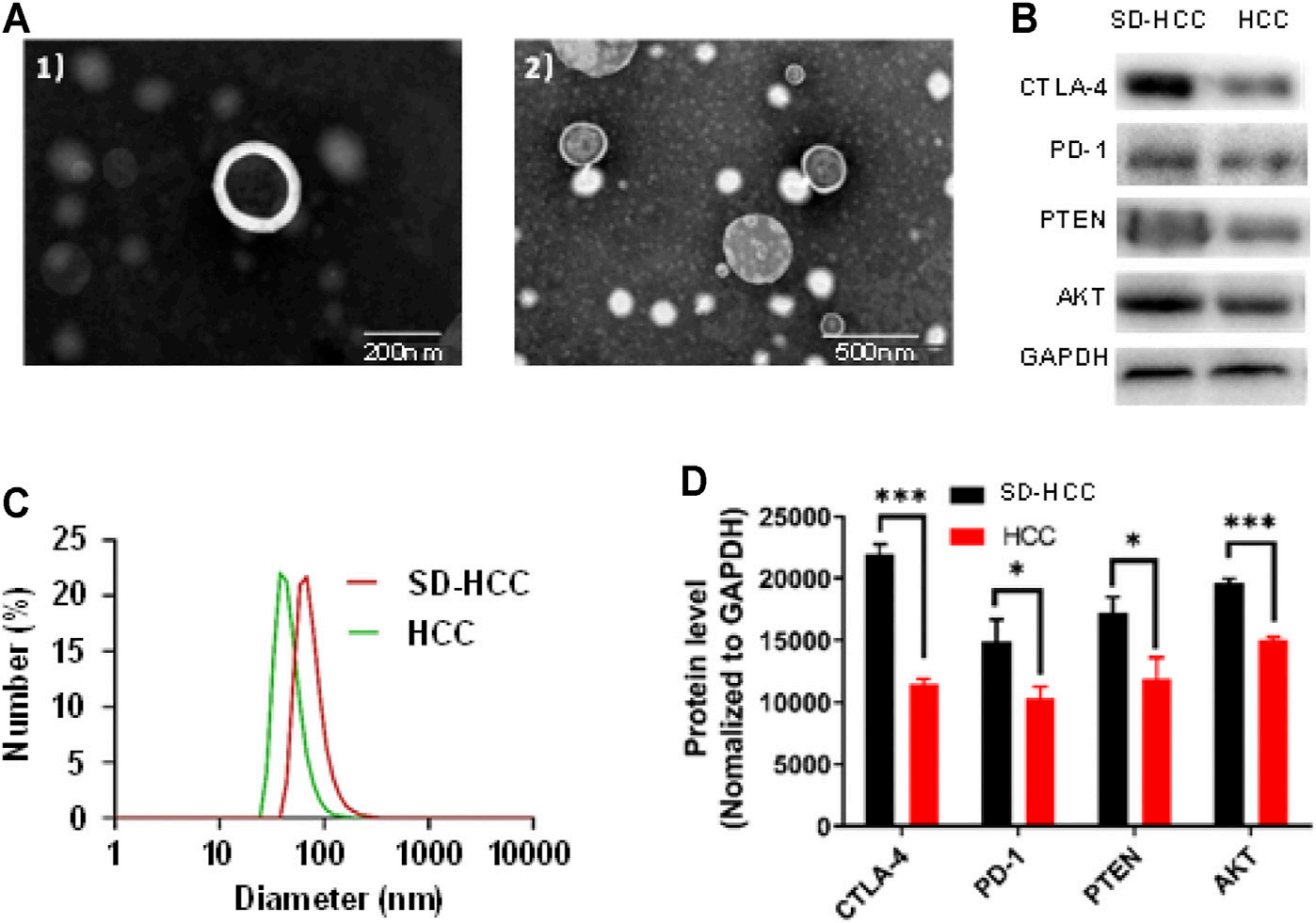
Exosome verification. **(A)** TEM images of 1) HCC exosomes, 2) SD-HCC exosomes; **(B)** exosomal protein level detection band chart; **(C)** size distribution of exosomes; **(D)** histogram of exosomal protein levels. *n* = 3, *p < 0.05, ***p < 0.001.

We chose mice from the HCC group and the SD-HCC group. After the preparation of the model was completed, blood was taken, and exosomes were extracted. We examined the morphology of HCC exosomes and SD-HCC exosomes by using transmission electron microscopy (TEM). The results displayed exosomes as spherical, membrane-bound vesicles (Figure 2A). The hydrodynamic diameter of the HCC and SD-HCC exosomes was measured to be 43 and 68 nm by dynamic light scattering (Figure 2C). Detecting the components of exosomes, we found that the levels of CTLA-4 and AKT in serum exosomes in SD-HCC were higher than those in the HCC group (*p* < 0.001). The contents of PD-1 and PTEN were higher than those in the HCC group, and the difference was significant (*p* < 0.05), suggesting that the exosomes were successfully extracted, and SD could affect the expression of CTLA-4 and other proteins in the exosomes (Figures 2B,D).

### Proliferation Inhibition

**FIGURE 3:**
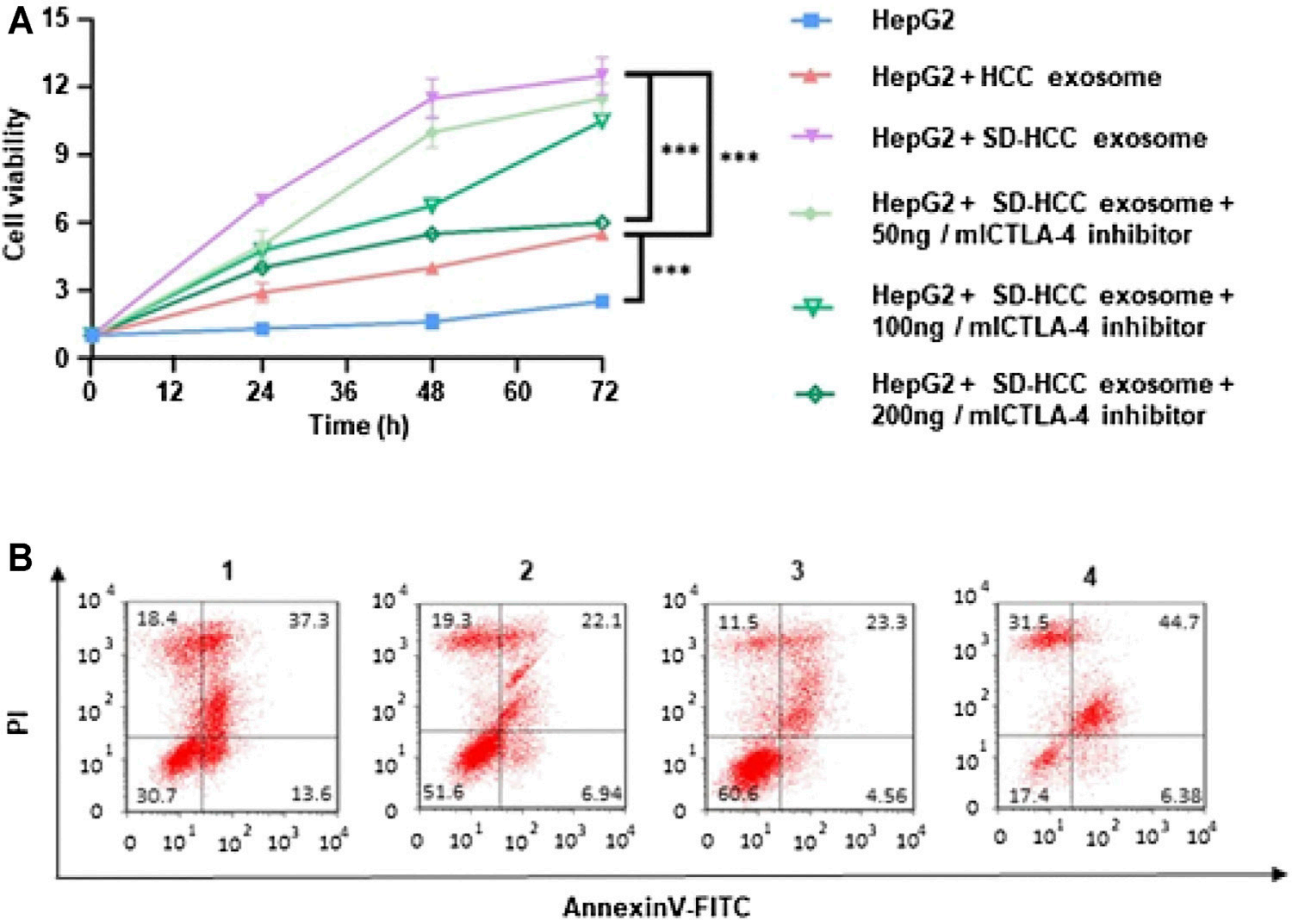
*In vitro* evaluation of cell proliferation of exosomes. **(A)** Relative cell viability of HepG2 after 72 h incubation of HCC exosomes and SD-HCC exosomes; **(B)** the Annexin V/PI apoptosis assay was measured by FCM analysis of HepG2 cells after being treated with each group for 24 h. *n* = 3, ^***^ *p* < 0.001. 1) HepG2, 2) HepG2 + HCC exosome, 3) HepG2 + SD-HCC exosome, 4) HepG2 + SD-HCC exosome + CTLA-4 inhibitor.

To explore the relationship between SD and cell proliferation *in vitro*, we generated cell growth curves evaluated by MTT. Briefly, 10 µl of PBS, liver cancer mouse serum, SD liver cancer mouse serum, SD liver cancer mouse serum plus CTLA-4 inhibitors were added separately into a 96-well plate with HepG2 cells. The results revealed that HCC cell proliferation was promoted by SD liver cancer mouse serum compared with liver cancer mouse serum in 48 and 72 h. The addition of CTLA-4 inhibitors could partially reverse this change (Figure 3A). Apoptosis study of HepG2 cells tested by flow cytometry showed that SD liver cancer mouse serum could induce least miniature cells apoptosis compared with liver cancer mouse serum. The addition of CTLA-4 inhibitors could partially reverse cell apoptosis (Figure 3B).

### Cell Migration and Invasion

**FIGURE 4:**
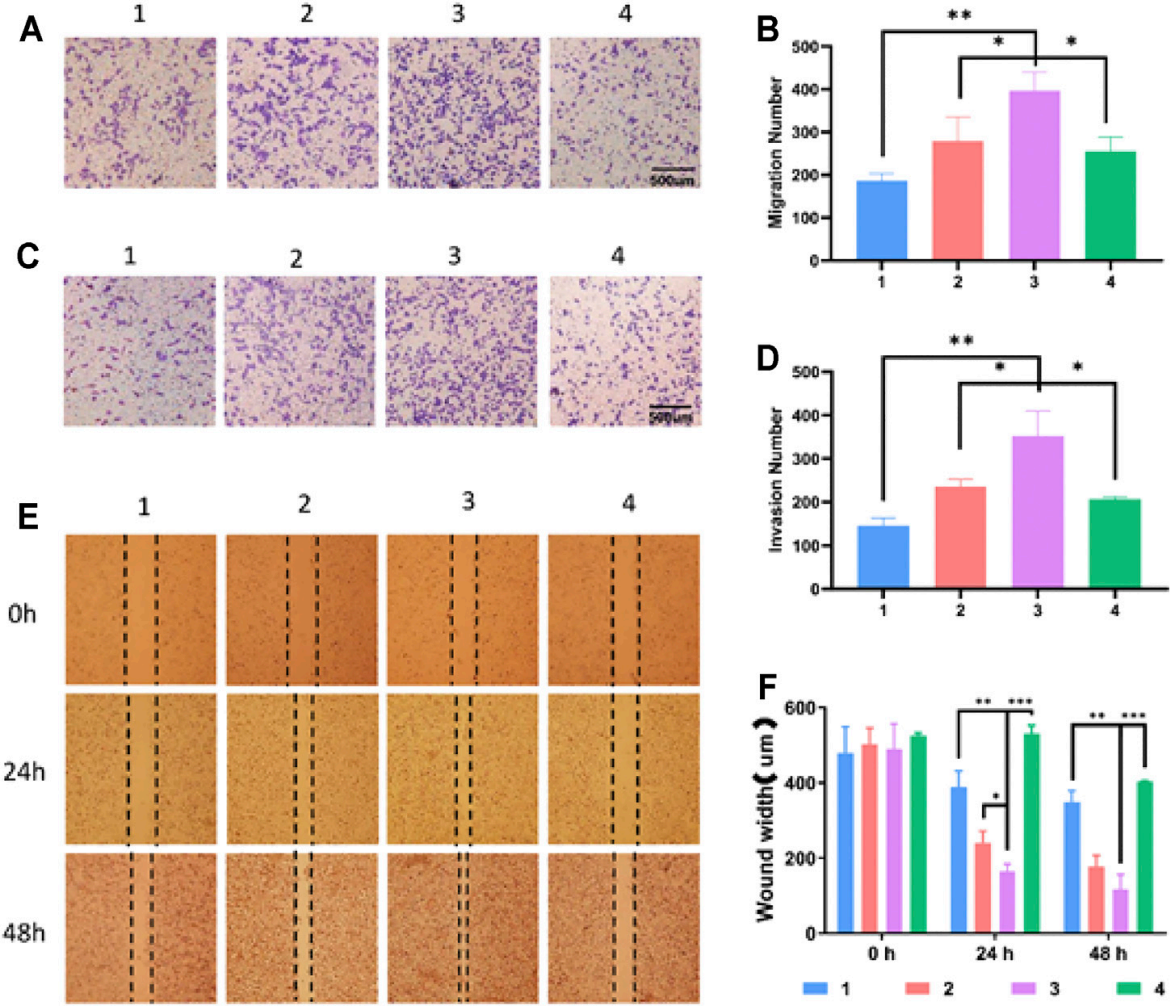
Effect of exosomes of SD on growth and invasion of HCC cells. **(A)** Cell migration experiment, **(B)** histogram of migration experiment statistics, **(C)** cell invasion test, **(D)** invasion experiment statistics histogram, **(E)** cell scratch test, **(F)** scratch experiment statistics histogram. *n* = 3, ^*^ *p* < 0.05, ^**^ *p* < 0.01,^***^ *p* < 0.001, 1) HepG2, 2) HepG2 + HCC exosome, 3) HepG2 + SD-HCC exosome, 4) HepG2 + SD-HCC exosome + CTLA-4 inhibitor.

To study the effect of SD on the migration and invasion of liver cancer cells, the HepG2 cells were treated with SD liver cancer mouse serum or liver cancer mouse serum for 48 h. The cell scratch method and transwell test were used to detect the migration and invasion ability of HepG2 cells after drug treatment. We found that compared with the HCC group and the SD-HCC add CTLA-4 inhibitor groups, the SD-HCC group had a stronger migration ability (Figures 4A,B) and stronger invasion ability (Figures 4C,D). In the scratch experiment, we observed that the diameter of the scratches in the SD-HCC group was narrower than in the other three groups at 24 and 48 h after the scratches were made. The differences are statistically significant (*p* < 0.001) (Figures 4E,F).

### H&E Staining of Liver Tumor

**FIGURE 5:**
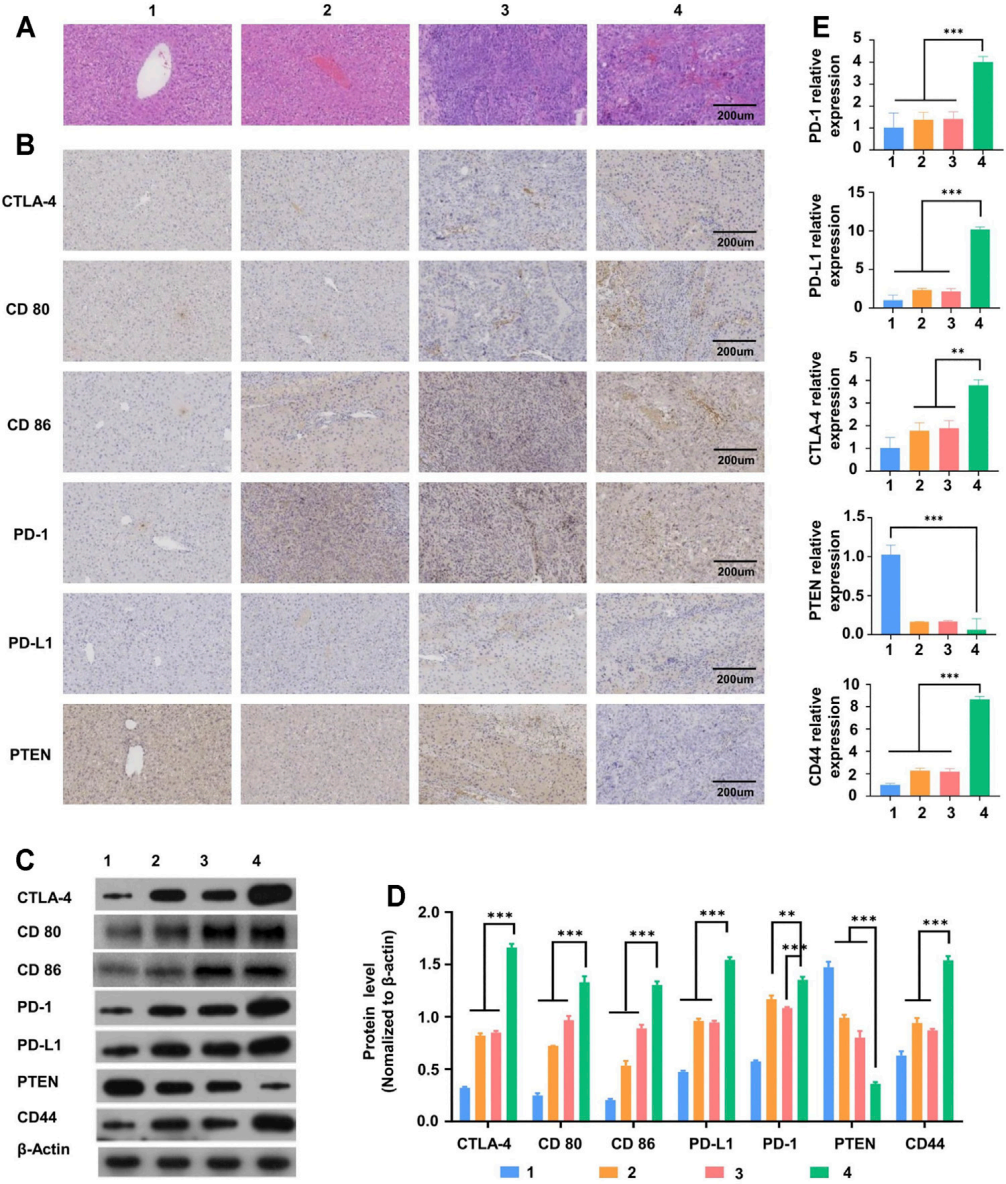
Liver tissue slice morphology and molecular mechanism of liver tumor occurrence and development. **(A)** H&E staining observation of morphological changes of liver tissue sections of mice in different treatment groups, **(B)** immunohistochemical detection of CTLA-4 and other immune checkpoint protein distribution, **(C)**Western blot detection of CTLA-4 and other immune checkpoint-related protein band diagram, **(D)** histogram, **(E)** RT-PCR detects the expression of mRNA related to immune checkpoints. *n* = 3, **p < 0.01,***p < 0.001, 1) control group, 2) SD group, 3) HCC group, 4) SD-HCC group.

Hematoxylin and eosin (H&E) staining of the liver tissues from the HCC and SD-HCC groups showed all sizes of carcinoma cells with some multinucleated giant tumor cells, and the funicular slices were distributed and accumulated irregularly without normal hepatocyte construction (Figure 5A). To some extent, the results indicated that the degree of malignancy of the tumor tissue was higher in the SD-HCC group than in the HCC group.

### Molecular Mechanism of Liver Tumor Occurrence and Development

In order to verify the influence of the internal environment of SD on liver tumor occurrence and development, we established liver cancer models and SD liver cancer models in the early stage. The results of immunohistochemistry and WB (Western Blot) showed that both the SD group and liver cancer group mice had significantly higher (*p* < 0.01) levels of CTLA-4 and PD-1 compared with the normal group. In addition, the liver cancer mice with SD had significantly higher levels of CTLA-4 and PD-1 proteins than those in the liver cancer group only (*p* < 0.01) (Figures 5B–D). The mRNA expression in tissues as analyzed by the RT-PCR test showed a similar trend (Figure 5E). All these results suggest that the internal environment of SD may affect liver tumor occurrence and development by disturbing CTLA-4 and other series of proteins and genes.

## Discussion

HCC is one of the most malignant cancers and is the third leading cause of cancer-related death due to its high recurrence and poor prognosis (Chen et al., 2016). HBV infection is a major risk factor for HCC, and Chinese HCC patients account for 50% of HCC patients in the world (Bray et al., 2018; Feng et al., 2019). Currently, treatment strategies for HCC patients include surgery, liver transplantation, chemotherapy, radiotherapy, biological therapy, and targeted molecular therapy, but the overall effect is not satisfactory due to the highly metastatic nature of HCC (Couri and Pillai, 2019). Therefore, it is necessary to search for reliable biomarkers and therapeutic targets to monitor the progress of HCC. The imbalance of the body’s internal environment is closely related to the occurrence and development of cancer, and genetic inheritance can only explain 5% of the occurrence of cancer (Bhattacharjee et al., 2013; Craig et al., 2020; Llovet et al., 2021). Eventually, changes in the body’s internal environment are unfavorable to the survival of normal cells but appropriate to the survival of tumor cells. “Bian Zheng Lun Zhi,” the core of TCM therapy systems, is based on syndrome differentiation, which helps to guide the design of individualized treatments (Ji et al., 2016; Zhang et al., 2020). Syndromes such as loose stools, abdominal distension after meals, loss of appetite, sallow complexion, weight loss, general weakness, and/or low disease resistance could be summarized as SD in TCM theoretical system. In the clinical investigation of 767 cases of gastric cancer patients, 32.86% of patients showed the syndrome of SD and stomach cold syndrome (Sun et al., 2010). SD syndrome is also considered to be the key pathogenesis of colorectal cancer (Sun et al., 2016a). It has been reported that traditional Chinese medicine for invigorating the spleen helps in delaying the pathological process of HCC cachexia induced by ascites by reducing the levels of IL-Lα, IL-6, and TNFα and inhibiting the activation of the ubiquitin–proteasome pathway (Sun et al., 2016b). Meanwhile, traditional Chinese medicine in invigorating the spleen could prolong the survival time and decrease tumor metastasis in the liver in mice, which may be associated with enhancing the expression of PTEN in the liver (Yin et al., 2008). Therefore, the internal environment of SD is especially vital for the occurrence and development of immune recognition disorders of HCC.

In our study, reserpine was used to establish a model of SD syndrome referring to the previous researches (Zhao et al., 2011; Luo et al., 2017). Chronic rather than acute doses of reserpine were used to induce the syndrome of SD in the mice in this study. In our analysis, we referred to each factor in the SD scores assessment (Luo et al., 2017) and found that the score of the SD group was higher than that of the control group. During the establishment of the model of SD syndrome, there were also significant differences between the SD group and the control group in the amount of daily food intake and the time to stop swimming. In addition, compared with the control group, the weight of the mice in the SD group decreased significantly, and this effect continued throughout the modeling process. The results of H&E staining showed that the malignant degree of tumor in the SD-HCC group was higher than that in the HCC group. These results indicated that the SD model was successfully established, and the factor of SD indeed affected the growth of the HCC mice.

In recent years, tumor immunotherapy has been the focus of research which has brought great benefits to cancer patients. However, due to a variety of complex factors, it is easy for tumors to escape from the host immune system during the treatment process, leading to treatment failure (Whiteside et al., 2011). Moreover, tumor immunotherapy also induces some serious toxic events and autoimmune diseases, such as pituitary hypophysitis, autoimmune hepatitis, pneumonia, and so on (Cogdill et al., 2017; Couzin-Frankel, 2017). Therefore, it is very necessary to find out the cancer-related immune mechanism. Exosomes (30–150 nm) are considered as messengers between cells, carrying a large number of macromolecules, including proteins, mRNAs, lipids, and miRNAs, which constitutes an important part of the tumor immune microenvironment (Wen et al., 2016; Bach et al., 2017; Ruivo et al., 2017; Su et al., 2021). Exosomes are nanoscale membranous vesicles secreted from intracellular multivesicular bodies (MVBs) or late endosomes into the extracellular space through extracellular action (Su et al., 2021). CD81, CD63, and TSG101 have become the most commonly used exosomal labeling proteins (Pegtel and Gould, 2019). In our study, after the establishment of the model, we selected the serum of the HCC group and SD-HCC group mice for separation of the exosomes. Exosomes were observed as spherical, membrane-bound vesicles by using TEM. The hydrodynamic diameter of the exosomes was measured as 43 and 68 nm by dynamic light scattering. These characteristics of exosomes are consistent with the results of other studies, which demonstrates that we successfully extracted exosomes of HCC. Studies have found that tumor-derived exosomes carry immunosuppressive proteins, such as PD-1, CTLA-4, FasL, TRAIL, etc. (Whiteside, 2013; Ukrainskaya et al., 2019; Benecke et al., 2021). Detecting the components of exosomes, we found the presence of PD-1, CTLA-4, PTEN, and AKT. Interestingly, the level of CTLA-4 of the SD-HCC group was the highest, which may indicate that CTLA-4 plays an important role in exosome HCC with SD syndrome.

CTLA-4 is highly expressed in lymphocytes CD4 + and CD8 + T (Phung et al., 2020), competing with the T-cell costimulator CD28 for binding to CD80 and CD86 with a higher affinity, which conveys inhibitory signals within the T cells (Van Coillie et al., 2020; Goenka et al., 2021). We examined liver tumor tissues to verify this relationship between CTLA-4 and T cells in the internal environment of SD. The results of immunohistochemistry and WB showed that CTLA-4, CD80, and CD86 were higher in the HCC and SD-HCC groups than in the other groups, and it was significantly higher in the SD-HCC group. The mRNA expression showed the same results. These data may indicate that T-cell activation and function were restricted. It has been reported that mutations in the CTLA-4 gene are associated with an increased risk of human autoimmune disorders (Ueda et al., 2003; Schubert et al., 2014). In immunotherapy of cancer, the use of blocking anti–CTLA-4 and anti–PD-1 antibodies have yielded promising results, and the 2018 Nobel Prize was granted to Jim P. Allison and Tasuku Honjo, who are the pioneers in this field (Wolchok, 2018). In the cell proliferation experiment, compared with the serum of HCC mice at 48 and 72 h, the serum of SD-HCC mice promoted the proliferation of HCC cells and the addition of CTLA-4 inhibitor could partially reverse this change. Detecting apoptosis on HepG2 cells showed that SD-HCC mice serum can induce the least cell apoptosis compared with liver cancer mouse serum and when added into CTLA-4 inhibitors could partially reverse cells apoptosis. In order to study the effect of SD on the migration and invasion of HCC cells, cell scratch assay and transwell test were used after drug treatment. Compared with the HCC group and the CTLA-4 inhibitor group, the SD-HCC group had stronger migration ability and stronger invasion ability. These results suggest that SD may boost the occurrence and development of liver cancer through exosome CTLA-4.

PTEN is an important tumor suppressor protein, and a loss in its function is associated with malignant transformation, progression, chemotherapy response, and survival of a variety of cancers (Su et al., 2016; Wang et al., 2018a; Luongo et al., 2019; Raffone et al., 2019). PTEN has an important effect on regulating the PI3K/Akt/mTOR signaling pathway, which plays an important role in the proliferation and maintenance of CSC self-renewal, according to reports (Su et al., 2016; Yang et al., 2016; Luongo et al., 2019). The relationship between PTEN and PI3K/Akt/mTOR can be partly illustrated in our study according to the result of detecting exosome components, which displayed the presence of PTEN and Akt in the exosomes and was higher in the SD-HCC group than in the HCC group. Growing evidence has indicated that PTEN has specific functions to interfere with stem/progenitor cells, including regulating the differentiation of neural SCs, promoting the differentiation of mesenchymal SCs, and maintaining the balance between proliferation and differentiation of hematopoietic SCs (Su et al., 2016). It is well known that CSCs have the characteristics of self-renewal and differentiation (Luongo et al., 2019; Nero et al., 2019; Cetintas and Batada, 2020). There are multiple biomarkers of CSCs, including CD133, SOX2, BMI1, CD44, Nanog, and ABCG2 (Hu et al., 2020). The PTEN/CD44 signaling pathway plays an important role in maintaining the characteristics of CSCs, involved in tumorigenesis, invasion and metastasis, and immune escape (Luongo et al., 2019; Nero et al., 2019; Cetintas and Batada, 2020). In this study, the expression of PTEN and CD44 is the highest in the SD-HCC group than in the other groups according to the results of immunohistochemistry, WB, and mRNA. We inferred that the SD factor could promote the expression of PTEN and CD44 and thus has a positive effect on the differentiation of CSCs. Besides liver cancer, the current treatments of various types of other cancers are also waiting for better outcomes. Although there are multiple forms of technologies those were explored, e.g., photoacoustic technology, reactive oxygen species, and cytotoxic peroxynitrite (ONOO^−^), the efficiency of cancer treatments is still far from satisfactory (Wang et al., 2021; Zhang et al., 2021). Here, our findings from exosomes may provide some hints from a new angle for high efficiency of treatment of various cancers.

In conclusion, in the HCC mouse model, we found that SD plays a key role in the occurrence and development of immune recognition disorders of HCC. In addition, at the molecular level, the exosome CTLA-4 expression in the SD-HCC group was upregulated, which played a powerful role in regulating the growth, self-renewal, and metastasis of HCC. SD also promoted the PTEN/CD44 pathway process. These important findings suggest that the internal environment of SD exosome CTLA-4 promotes the metastasis of liver cancer by regulating the PTEN/CD44 pathway. However, the molecular mechanism of how CTLA-4 regulates the PTEN/CD44 pathway remains unclear and needs further study.

## Data Availability

The original contributions presented in the study are included in the article/Supplementary Material, further inquiries can be directed to the corresponding authors.
