# Supplementary material for: Exosome CTLA-4 Regulates PTEN/CD44 Signal Pathway in Spleen Deficiency Internal Environment to Promote Invasion and Metastasis of Hepatocellular Carcinoma
Source: Front Pharmacol. 2021 Oct 20;12:757194. doi: 10.3389/fphar.2021.757194 (PMC8564353; doi:10.3389/fphar.2021.757194)
Supplement: Supplementary file 1 [file Table1.docx]

**The sequences of the primers**

| Genes | Primer | Sequences |
| --- | --- | --- |
| PD-1  PD-1 | Forward | CTCCAAGACATGAGGATGGAC |
|  | Reverse | CCTGAAGTGAGCTCTCACTC |
| PD-L1 | Forward | GATGGAGTCATGAGTGTTGAAG |
| PD-L1 | Reverse | GAGTCAGACAGCAAGAGCCTG |
| CTLA-4 | Forward | GAGCTATGTGACATAGCCAGTG |
| CTLA-4 | Reverse | GACCTCGAGTCCAACCTGATG |
| PTEN | Forward | GTCTTAAGTGGCTTGTGTGTGG |
| PTEN | Reverse | GCTTACATCCTGATACCTGTTG |
| CD44 | Forward | CTATATGCAGCAAGCCACTC |
| CD44 | Reverse | CAGAATCATCACCACTATGGC |
